# A fast and sensitive activity assay for lytic polysaccharide monooxygenase

**DOI:** 10.1186/s13068-018-1063-6

**Published:** 2018-03-23

**Authors:** Erik Breslmayr, Marija Hanžek, Aoife Hanrahan, Christian Leitner, Roman Kittl, Božidar Šantek, Chris Oostenbrink, Roland Ludwig

**Affiliations:** 1 Department of Food Science and Technology, Vienna Institute of Biotechnology, BOKU-University of Natural Resources and Life Sciences, Muthgasse 18, 1190 Vienna, Austria; 2 Department of Material Science and Process Engineering, Institute of Molecular Modeling and Simulation, BOKU-University of Natural Resources and Life Sciences, Muthgasse 18, 1190 Vienna, Austria; 3 Department of Biochemical Engineering, Faculty of Food Technology and Biotechnology, University of Zagreb, Pierottijeva 6, 10000 Zagreb, Croatia

**Keywords:** Activity assay, 2,6-Dimethoxyphenol, Hydrogen peroxide, Biomass degradation, Lytic polysaccharide monooxygenase, Peroxidase activity

## Abstract

**Background:**

Lytic polysaccharide monooxygenases (LPMO) release a spectrum of cleavage products from their polymeric substrates cellulose, hemicellulose, or chitin. The correct identification and quantitation of these released products is the basis of MS/HPLC-based detection methods for LPMO activity. The duration, effort, and intricate analysis allow only specialized laboratories to measure LPMO activity in day-to-day work. A spectrophotometric assay will simplify the screening for LPMO in culture supernatants, help monitor recombinant LPMO expression and purification, and support enzyme characterization.

**Results:**

Based on a newly discovered peroxidase activity of LPMO, we propose a fast, robust, and sensitive spectrophotometric activity assay using 2,6-dimethoxyphenol (2,6-DMP) and H_2_O_2_. The fast enzymatic assay (300 s) consists of 1 mM 2,6-DMP as chromogenic substrate, 100 µM H_2_O_2_ as cosubstrate, and an adequate activity of LPMO in a suitable buffer. The high molar absorption coefficient of the formed product coerulignone (*ε*_469_ = 53,200 M^−1^ cm^−1^) makes the assay sensitive and allows reliable activity measurements of LPMO in concentrations of approx. 0.5–50 mg L^−1^.

**Conclusions:**

The activity assay based on 2,6-DMP detects a novel peroxidase activity of LPMO. This activity can be accurately measured and used for enzyme screening, production, and purification, and can also be applied to study binding constants or thermal stability. However, the assay has to be used with care in crude extracts, because other enzymes such as laccase or peroxidase will interfere with the assay. We also want to stress that the peroxidase activity is a homogeneous reaction with soluble substrates and should not be correlated to heterogeneous LPMO activity on polymeric substrates.
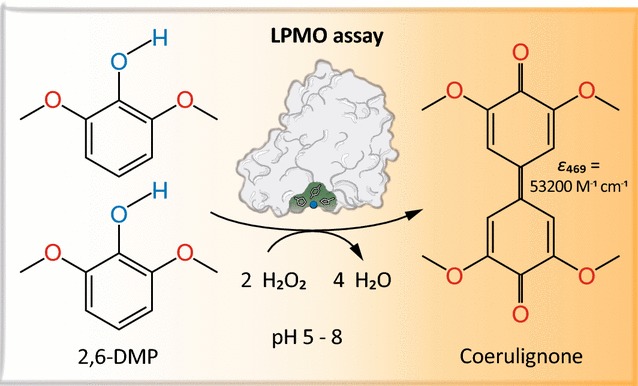

**Electronic supplementary material:**

The online version of this article (10.1186/s13068-018-1063-6) contains supplementary material, which is available to authorized users.

## Background

Lytic polysaccharide monooxygenases (LPMO; CAZy auxiliary activities AA9, AA10, AA11, and AA13 [1]; preliminary EC classification 1.14.99.B6) catalyze the oxidative depolymerization of diverse polymeric carbohydrates such as cellulose (AA9, AA10), hemicellulose (AA9, AA14 [2]), chitin (AA10, AA11), and starch (AA13). The polymeric nature of these substrates and the wide spectrum of released products make the assessment of LPMO activity difficult. Typically, LPMO and a polymeric substrate are incubated under defined assay conditions in vials for a defined period (up to 1 day) before a calibrated HPLC method is used for the quantitation of the products. If a new LPMO/substrate combination is measured, the reaction products have to be identified by HPLC standards or mass spectrometry. Protocols for the determination of reaction products were published for cellulose (HPAEC and MALDI-TOF [3, 4]; PGC, CAD and ESI-MS [5]), hemicellulose (HPAEC and ESI-MS [6]; HPAEC and MALDI-TOF, [7]), and chitin (UHPLC-HILIC coupled to MALDI-TOF, [8, 9], and RP-UHPLC-MS [10]). HPLC-MS measurements of LPMO activity give detailed insight into the formed reaction products, but are time consuming. The detection of reaction products is restricted to low molecular weight cleavage products (DP 1–6), which are soluble in the reaction buffer and the HPLC eluent, and for which HPLC standards are available. The quantitation of larger products that remain in the insoluble fraction can be achieved by total hydrolysis. By this way, C1-oxidized products have been quantified by HPLC, but the quantification of C4-oxidized products remains a struggle. Also, the most commonly used cellulosic substrate is phosphoric acid swollen cellulose (PASC), which is not a physiological substrate of LPMO. Nevertheless, if suitable reaction conditions, sampling time points and correct calibration data for the detected species are used, HPLC-based methods provide the most accurate results of physiological LPMO activity. NMR has also been used to follow the action of LPMO on polymeric substrates [4, 6, 11, 12], but its strength lies in the identification of reaction products and not in the measurement of enzymatic activity.

An activity measurement of LPMO on PASC, analyzed with polysaccharide analysis by carbohydrate gel electrophoresis (PACE), has been reported to yield mostly cellobiose and cellotriose [13]. The PACE method allows the use of natural substrates and multiple samples and gives semi-quantitative results; however, the effort for preparation and measurement is much higher compared to a spectrophotometrical activity assay. It was also shown that the used C4-oxidizing LPMO is able to cleave cellotriose, which demonstrates that LPMOs can act on small oligosaccharides and perform successive depolymerization reactions. This feature of some LPMOs additionally makes an absolute quantitation of reaction products difficult. The use of derivatized cello-oligosaccharides as a substrate to follow cleavage by FRET quenching is far less affected by successive depolymerization. This method was used to determine kinetic constants of LPMO for a fluorescence-labeled cellotetraose [13] and is certainly qualified to measure homogeneous LPMO activity when the substrate becomes available. A microarray method to detect LPMO activity on hemicelluloses was established and gave semi-quantitative results [7]. A microplate-based detection of insoluble C1-oxidized products of LPMO was achieved by fluorescence-labeling with 7-amino-1,3-naphthalene-disulfonic acid (ANDA). The method was verified by X-ray photoelectron spectroscopy [14]. Finally, chromogenic polysaccharide hydrogel substrates were used to screen for the activity of different carbohydrate-active enzymes including LPMO. This semi-quantitative method can detect the cleavage of specific oligosaccharides by LPMO and can be used to screen for its activity on agar plates [15].

In contrast to these methods, which all detect the more or less natural activity of LPMO, stands a method which measures LPMO activity in a spectrophotometric/-fluorimetric assay based on the release of H_2_O_2_, which is a product of the uncoupling reaction of the activated copper–oxygen complex in the absence of substrate. The Amplex red assay is a coupled enzyme assay [16], which uses the formation of hydrogen peroxide by LPMO in the presence of oxygen and a reductant like ascorbic acid or cellobiose dehydrogenase (CDH) for the peroxidase-catalyzed oxidation of Amplex red to resorufin which can be detected spectrophotometrically (*ε*_571_ = 58,000 M^−1^ cm^−1^) or fluorimetrically (Ex = 569 nm/Em = 585 nm). This assay can be used to detect the production and purification of LPMOs; however, the uncoupling reaction is slow, the sensitivity is low, and the assay requires a high LPMO concentration (20–574 mg L^−1^) for reliable measurements. It is also affected by metal ions present in fermentation media, which requires re-buffering in spin columns or diafiltration. The Amplex red-based assay is most useful for quick activity measurements to quantify LPMO activity during protein purification. It is not suited to screen for LPMO activity in culture supernatants, to characterize mutational changes, or to measure physiological reactions. Despite its shortcomings, the Amplex red-based assay has been widely used and shows the need for a fast spectrophotometric assay to screen, produce, and purifiy LPMO.

**Fig. 1:**
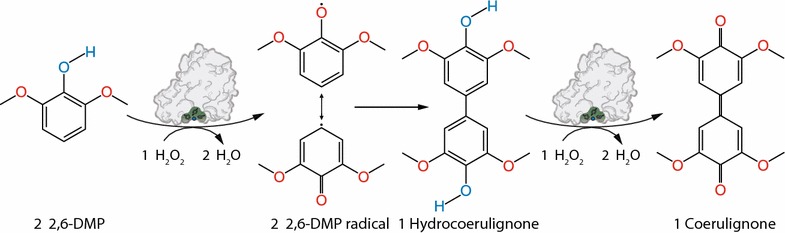
2,6-DMP oxidation. LPMO catalyzes the oxidation of 2,6-DMP to the corresponding phenoxy radical at the expense of H_2_O_2_. The active-site Cu(II) is reduced by 2,6-DMP which generates the 2,6-DMP radical. Two formed 2,6-DMP radicals dimerize rapidly to hydrocoerulignone, which again is quickly converted to coerulignone by LPMO. The stoichiometry of the peroxidase reaction is 1:1

In this work, we describe a new reaction of LPMO, which was found after H_2_O_2_ was demonstrated to be a LPMO cosubstrate [17]. Although the reaction mechanism remains to be fully elucidated, the published results show a highly increased activity of LPMO in the presence of H_2_O_2_, which outperforms oxygen as cosubstrate in the tested reactions. This peroxygenase-like reaction depends on the initial reduction of the active-site copper from its resting state Cu(II) to Cu(I) by a reductant like ascorbic acid, before binding the cosubstrate H_2_O_2_ [18]. Using H_2_O_2_ as cosubstrate, it was tried to find a homogeneous reaction to assess LPMO activity in solution. Several chromogenic substrates were tested, the best selected, assay conditions optimized, and the established LPMO assay was validated. The reaction of the developed assay is shown in Fig. 1. The substrate 2,6-dimethoxyphenol (2,6-DMP) and the cosubstrate H_2_O_2_ are converted to the 2,6-DMP radical and water. Then two 2,6-DMP phenoxy radicals dimerize and form hydrocoerulignone, which is again oxidized by LPMO to the chromogenic product coerulignone. In addition to assay LPMO’s peroxidase activity during enzyme production and purification, the developed assay can also be used to determine binding constants or the thermal stability of LPMOs. The results obtained with three LPMOs indicate that the assay should be broadly applicable.

## Results and discussion

### Screening for chromogenic substrates

**Fig. 2:**
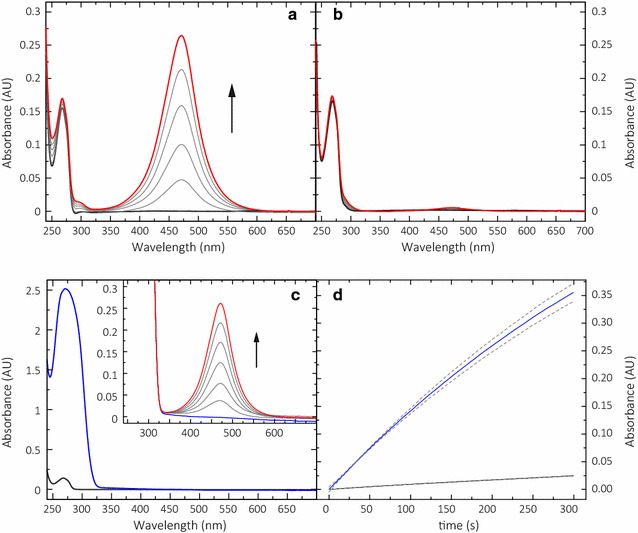
Conversion of 200 µM 2,6-DMP and hydrocoerulignone by 2 µM *Nc*LPMO9C in 100 mM sodium succinate/phosphate buffer, pH 6.0. **a** Reaction of 2,6-DMP in the presence of 100 µM H_2_O_2_ and 250 µM dissolved O_2_, and **b** in the presence of 250 µM O_2_ only, followed for 5000 s. Time frequency between each trace is 1000 s. **c** Spectra of 2,6-DMP (black line) and hydrocoerulignone (blue line), 200 µM each. Inset demonstrates the conversion of hydrocoerulignone; note that the occurring peak is similar to **a**, followed for 300 s. Time frequency between each trace is 50 s. **d** Time course of the *Nc*LPMO9C (0.3 µM)-catalyzed conversion of 1 mM 2,6-DMP (black line) and 1 mM hydrocoerulignone (blue line) measured at 469 nm in the presence of 100 µM H_2_O_2_ in 100 mM sodium succinate/phosphate buffer, pH 6.0. The data are expressed as mean values (± SD), from three independent repeats

Various substituted mono-, di-, and triphenols were tested with *Nc*LPMO9C from *Neurospora crassa* to find a chromogenic reaction in the presence of H_2_O_2_ (Additional file 1). From these, color formation was observed for gallic acid, pyrochatecol, and sinapic acid (Additional file 2), but the fastest increase in absorption was observed for 2,6-DMP (Fig. 2a). When O_2_ is used as a cosubstrate, only a very slow reaction with 2,6-DMP is observed (Fig. 2b). The reaction with 100 µM H_2_O_2_ is 62 times (at pH 6.0) or 56 times (at pH 7.5) faster than with 250 µM O_2_. The formation of the 2,6-DMP phenoxy radical and the proposed dimerization to hydrocoerulignone [19] cannot be observed spectrophotometrically, but the final reaction product coerulignone is strongly colored and the peak wavelength at 469 nm (Fig. 2c) is little obstructed by matrix components of cultivation media. The final product of the LPMO-catalyzed reaction starting from either 2,6-DMP or hydrocoerulignone is spectroscopically identical. Therefore, it was concluded that hydrocoerulignone is a reaction intermediate as shown in Fig. 1.

### Buffer and pH effects on LPMO activity

**Fig. 3:**
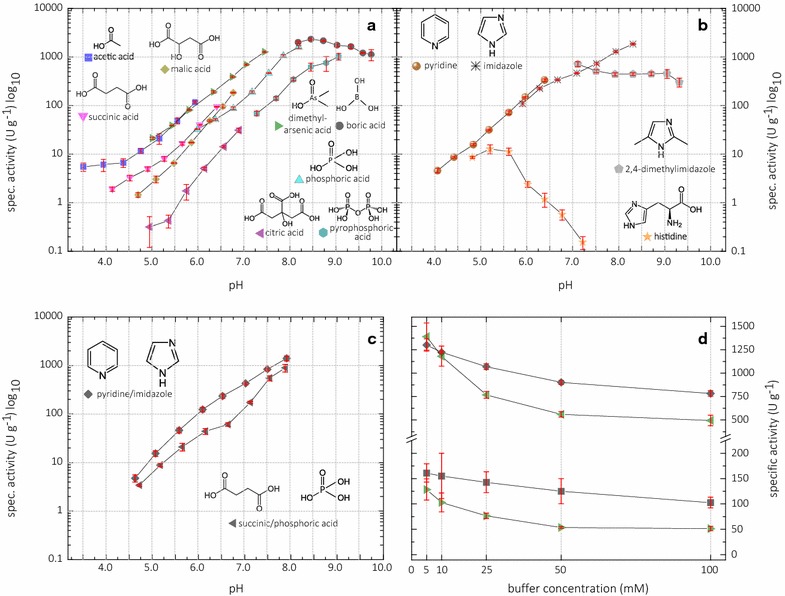
pH-dependent activity of *Nc*LPMO9C (0.10 – 3.75 µM; 30 µM for histidine chloride buffer) in various buffers with 25 mM 2,6-DMP and 100 µM H_2_O_2_. **a** 100 mM sodium carboxylate, arsenate, phosphate, and borate buffers. **b** 100 mM imidazole or pyridine chloride buffers. **c** Anionic and cationic mixed buffers spanning a broader pH range. **d** Sensitivity of the *Nc*LPMO9C-catalyzed reaction to ionic strength in the cationic broad range buffer (squares) and the anionic broad range buffer (triangles) at pH 6.0 (lower traces) and 7.5 (higher traces). The data are expressed as mean values (± SD), from three independent repeats

After finding 2,6-DMP as a suitable chromogenic substrate, the reaction conditions for *Nc*LPMO9C were optimized. A screening of different buffers showed that 2,6-DMP conversion increases monotonically between pH 4 and 8. The gain in activity was so high that a logarithmic scale had to be used for the pH profile (Fig. 3). The molar absorption coefficient of the chromogenic product coerulignone does not change in this pH range. Mono-, di-, and tricarboxylic or hydroxycarboxylic acids were used to buffer the reaction between pH 3.5 and 7.0. The observed activity is correlated to the number of carboxy and hydroxy groups present in the buffering molecule and decreases in the following order: acetate > succinate > malate > citrate (Fig. 3a). Citrate is by far the worst buffer resulting in a 40-fold lower activity at pH 6.0 than acetate. This might be caused by the chelating properties of bi- or trichelate anions, which potentially interact with the active-site copper in LPMO. At pH values from 6.0 to 8.0, dimethyl arsenate and phosphate are good buffers as boric acid is for pH values from 8.0 onwards. A similar effect as for carboxylic acids is observed for dimethyl arsenate > phosphate > pyrophosphate. More hydroxyl and oxy groups decrease the activity of LPMO 3- and 15-fold when compared to dimethyl arsenate at pH 7.5.

Cationic buffer based on pyridine, imidazole, and 2,4-dimethylimidazole result in LPMO activities as high as in the best anionic buffer (Fig. 3b). The pH profile of histidine, a zwitterionic species, is the only one which is monotonically decreasing between pH 5.5 and 7.0, indicating that the deprotonation of the imidazole ring inhibits LPMO activity, possibly by coordination of the active-site copper atom. Based on these results, it is concluded that many buffers can be used for the assay. We suggest the non-harmful sodium succinate/phosphate buffer with a high buffer capacity over a broad pH range of 4.5–8.0 (Fig. 3c).

As already indicated by the results with di- and tricarboxylic acids, the ionic strength of the buffer also influences the LPMO activity (Fig. 3d). The low ion concentration in 5 mM buffers lead to a twofold increase of activity over the 100 mM buffers either at pH 6.0 or 7.5. However, further experiments were performed with the 100 mM sodium succinate/phosphate buffer to ensure a high buffer capacity in the activity assay.

A problem observed in 2,6-DMP-based activity assays is the reaction of 2,6-DMP radicals with the final, colored product coerulignone, which decreases the absorbance and thereby affects the assay. This effect is also observed in laccase and peroxidase assays, but rarely covered in publications. This reaction cannot be stopped, but suppressed by using low 2,6-DMP concentrations and a low enzymatic activity in the assay. We suppose that a high concentration of the formed 2,6-DMP radical intermediates is responsible for the subsequent decolorization of coerulignone possibly by a polymerization reaction [20–23]. On the other hand, higher 2,6-DMP concentrations increase the reaction rate of LPMO. Therefore, various 2,6-DMP concentrations at pH 6.0 and 7.5 were examined to find an appropriate range. The applicable 2,6-DMP concentration varies with pH and lies between 1–25 mM at pH 6.0 and 0.5–2 mM at pH 7.5. The 2,6-DMP concentration of 1 mM was used as a compromise to achieve reasonable enzymatic activity and assay stability. In cases where the assay should be as sensitive as possible, the 2,6-DMP concentration can be increased to the pH-dependent upper limit, but a shorter assay time has to be chosen to avoid the faster onset of the coerulignone decolorization reaction. When the assay is established for a new LPMO, it is important to evaluate this possible influence of the decolorization reaction. The assay is not affected when the increase of absorbance at 469 nm under steady-state conditions is strictly linear. A way to make the assay more sensitive would be the usage of hydrocoerulignone instead of 2,6-DMP, which, at a 1 mM substrate concentration has 15 times faster reaction rate (Fig. 2d). Unfortunately, hydrocoerulignone is difficult to solubilize in water. The faster hydrocoerulignone conversion also indicates that the 2,6-DMP oxidation is the rate limiting step in the assay and ensures that the assay measures the oxidation rate of 2,6-DMP.

### Absorption coefficient and stoichiometry

**Fig. 4:**
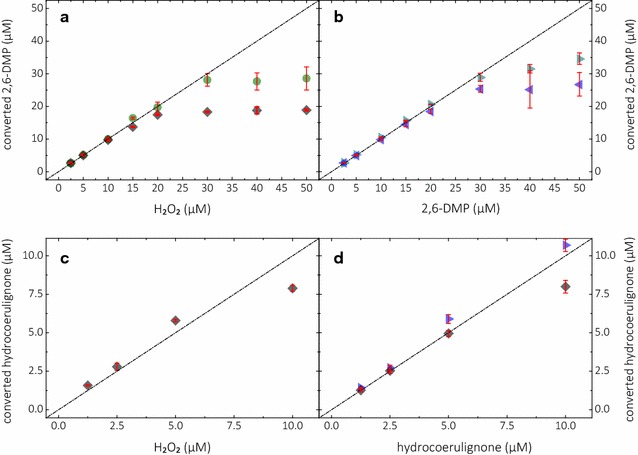
Reaction stoichiometry. Calculated concentrations of converted 2,6-DMP (top graphs) or hydrocoerulignone (bottom graphs) after oxidation with *Nc*LPMO9C, laccase, and horseradish peroxidase. Experiments with setting different H_2_O_2_ concentrations (left graphs) or setting different concentrations of 2,6-DMP or hydrocoerulignone (right graphs). **a** Linear increase of converted 2,6-DMP at pH 7.5 until 20 µM for *Nc*LPMO9C (black diamonds) or 30 µM for horseradish peroxidase (green circle). **b** Linear increase of converted 2,6-DMP at pH 6.0 (blue triangle) and pH 7.5 (cyan triangles) until 30 µM for laccase without addition of H_2_O_2_. **c** Linear increase of converted hydrocoerulignone at pH 6.0 and **d** at pH 7.5 until 5 µM for *Nc*LPMO9C (black diamonds; with addition of 100 µM H_2_O_2_) and pH 6.0 for laccase (blue triangle; without addition of H_2_O_2_). The data are expressed as mean values (± SD), from at least three independent repeats. Linear range was taken to calculate the molar absorption coefficient for coerulignone (*ε*_469_ = 53,200 M^−1^ cm^−1^). Dashed line represents the ideal 1:1 stoichiometry for the 2,6-DMP:H_2_O_2_ ratio based on the molar absorption coefficient

The molar absorption coefficient of the chromogenic product coerulignone was determined to be *ε*_469_ = 53,200 M^−1^ cm^−1^ between pH 4.0 and 8.0 and validated for pH 6.0 and 7.5 by titration experiments with horseradish peroxidase, LPMO, and laccase (Fig. 4a, b). This determined molar absorption coefficient deviates slightly from the previously determined and commonly used absorption coefficients for peroxidase, peroxygenase, and laccase activity assays: *ε*_469, 2,6-DMP_ = 27,500 M^−1^ cm^−1^ at pH 3.0, 4.5, and 5.0 [24–27] and *ε*_469, coerulignone_ = 49,600 M^−1^ cm^−1^ at pH 4.5 [19, 28–30]. The molar absorption coefficient of 27,500 M^−1^ cm^−1^ was reported for 2,6-DMP and was calculated by dividing the coefficient for coerulignone (55,000 M^−1^ cm^−1^) by the stoichiometry of 2,6-DMP:coerulignone = 2:1. However, we strongly suggest to assign the molar absorption coefficient to the actual chromogenic substance coerulignone.

The stoichiometry of the assayed LPMO reaction was determined in spectrophotometric cuvettes by following the conversion of substrate and cosubstrate in various concentrations. To this purpose, we assumed first the reaction stoichiometry to be similar to horseradish peroxidase, which is reported to be 1:1 for 2,6-DMP and H_2_O_2_. The same reaction stoichiometry for the LPMO-catalyzed conversion of 2,6-DMP and H_2_O_2_ has been confirmed by comparing the obtained results with horseradish peroxidase as catalyst (Fig. 4a). Because this LPMO reaction is similar to the peroxidase-catalyzed reaction, we call it peroxidase activity, until investigated in more detail. It was found that two 2,6-DMP molecules undergo a one-electron oxidation into the corresponding phenoxy radicals and donate in total two electrons to LPMO, which are transferred upon the acceptor hydrogen peroxide (Fig. 1). Up to a conversion of 20 µM 2,6-DMP, the measurements with LPMO and horseradish peroxidase (the reference reaction) correlate perfectly with the 2,6-DMP:H_2_O_2_ stoichiometry of 1:1 (Fig. 4a), but deviate from it at higher 2,6-DMP concentrations because of the coerulignone decolorization reaction described above. Figure 3b shows 2,6-DMP conversion by laccase at pH 6.0 and 7.5 (without H_2_O_2_), which also correlates well with the data in Fig. 3a. The two 2,6-DMP radicals dimerize into one hydrocoerulignone molecule, which is spectroscopically difficult to discriminate from 2,6-DMP. Hydrocoerulignone also reacts with H_2_O_2_ in an LPMO-catalyzed reaction (Fig. 4c, d) or with laccase in the absence of H_2_O_2_ (Fig. 4d) and forms the chromogenic product coerulignone with a stoichiometric ratio of 1:1.

### Kinetics of the LPMO reaction

The peroxidase activity was found for three different LPMOs from two different organisms. The specific activity of purified enzyme preparations was measured at standard conditions: *Neurospora crassa* LPMO9C: 32.3 ± 0.9 U g^−1^, LPMO9F: 2.2 ± 0.2 U g^−1^, and *Myriococcum thermophilum* LPMO (gene identifier Myrth2p4_000359): 30.9 ± 0.7 U g^−1^. The specific activity between these homogeneously purified LPMOs differs, which indicates a different peroxidase activity of the enzymes, but it demonstrates that this activity is present in at least some LPMOs. Bissaro et al. have shown that LPMO activity with H_2_O_2_ is concentration dependent [17] and Kuusk et al. derived a *K*_M_ value of CBP21 for H_2_O_2_ which was 2.8 ± 1.3 µM [18]. The different measured specific activities of the three LPMOs can therefore also indicate different affinities towards the cosubstrate.

**Fig. 5:**
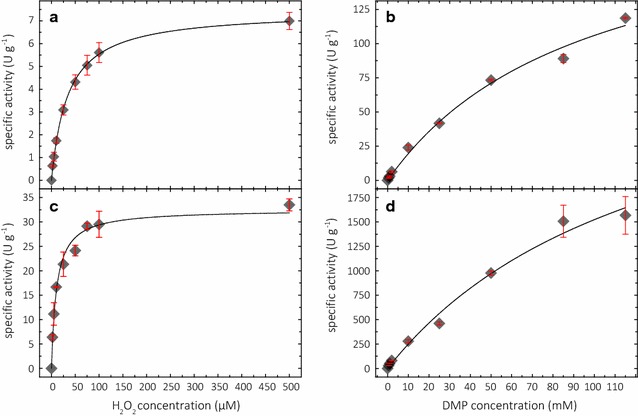
Michaelis–Menten kinetics at pH 6.0 (**a**, **b**) and pH 7.5 (**c**, **d**). For H_2_O_2_ kinetics: graphs with different H_2_O_2_ concentrations (**a**, **c**) and 1 mM 2,6-DMP. For 2,6-DMP kinetics: graphs with different 2,6-DMP concentrations (**b**, **d**) and 100 µM H_2_O_2_

To investigate the dependence of the reaction rate on the substrate and cosubstrate concentration, the apparent kinetic constants of *Nc*LPMO9C for both substrates were determined. The affinity for 2,6-DMP is very low and no saturation was obtained. From the measurements, a *K*_M_ > 100 mM was extrapolated, which shows that the LPMO active site is not evolved to bind 2,6-DMP. Laccases, which have a binding-site for phenolic substrates, have reported *K*_M_ values for 2,6-DMP in the range of 15–1000 µM and some outliers higher than these values [31]. For peroxidases, *K*_M_ values in the range of 8 µM for manganese peroxidase, 73 µM for dye-decolorizing peroxidase, and 80 µM for versatile peroxidase [32–34] are reported. The oxidation potential of 2,6-DMP was measured by cyclic voltammetry (Additional file 3). At a 1 mM 2,6-DMP concentration, the onset potential is 150 mV vs. Ag|AgCl at pH 6.0 and 25 mV at pH 8.0. The published midpoint potentials for LPMOs are around 100 mV vs. Ag|AgCl [35]. At increasing 2,6-DMP concentrations, the oxidative onset potential decreases. These effects might add to the higher observed activity at higher substrate concentrations and at higher pH. Compared to 2,6-DMP, the cosubstrate H_2_O_2_ binds strongly to LPMO and apparent *K*_M_ values of *Nc*LPMO9C for H_2_O_2_ at pH 6.0 and 7.5 are between 7.3 and 41.1 µM (Table 1, Fig. 5) which compare well to the value reported by Kuusk et al. [18]. We are not aware of a reported apparent *K*_M_ value of an LPMO for oxygen, but the *K*_M_ values obtained for H_2_O_2_ are 6- to 34-fold lower than the O_2_ concentration in air saturated, aqueous buffers.

**Table 1:** Kinetic constants of *Nc*LPMO9C for H_2_O_2_ and 2,6-DMP

	Kinetic constants of *Nc*LPMO9C for H_2_O_2_			Kinetic constants of *Nc*LPMO9C for 2,6-DMP		
	Cosubstrate 2,6-DMP (mM)	*K*_M_ (µM)	*V*_max_ (U g^−1^)	Cosubstrate H_2_O_2_ (µM)	*K*_M_ (mM)	*V*_max_ (U g^−1^)
pH 6.0	1	34.3 ± 2.4	7.5 ± 0.2	25	245 ± 74	270 ± 20
	10	39.8 ± 2.5	32.6 ± 0.8	100	100 ± 13	235 ± 15
	25	41.1 ± 2.7	59.5 ± 1.4	300	144 ± 28	352 ± 43
pH 7.5	0.5	7.3 ± 0.9	22.3 ± 0.6	25	172 ± 49	2500 ± 400
	1.0	10.7 ± 1.1	32.5 ± 0.8	100	132 ± 32	2250 ± 250
	2.0	11.8 ± 0.6	50.2 ± 1.1	300	124 ± 31	2300 ± 300

The constants were determined for pH 6.0 and 7.5 and for three different cosubstrate concentrations. It is obvious that for 2,6-DMP no saturation was achieved and therefore the kinetic constants for H_2_O_2_ were not determined under pseudo-first-order conditions

We also found no significant reduction of 2,6-DMP conversion when oxygen was removed from the LPMO assay. The observed specific LPMO activity in the presence of H_2_O_2_ and O_2_ was 32.3 ± 0.9 U g^−1^, whereas in the glove box under anaerobic conditions a specific activity of 30.6 ± 1.8 U g^−1^ was obtained.

### Stability of the assay and control experiments

**Fig. 6:**
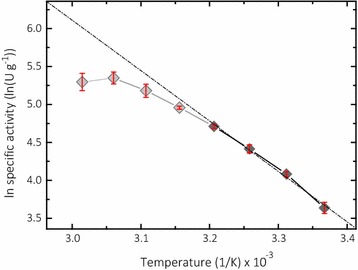
Arrhenius plot of *Nc*LPMO9C activity measured at different temperatures. Higher temperatures show an off leveling in activity, which corresponds from instability of the assay and further from inactivation of LPMO. Gray diamonds were not taken for calculation of the linear fit and activation energy. The data are expressed as mean values (± SD), from four independent repeats

To assess the stability of the assay components, the LPMO assay was performed at different temperatures between 25 and 60 °C (Fig. 6). The Arrhenius plot shows an almost linear increase between 25 and 40 °C. The assay components including *Nc*LPMO9C are relatively stable in this range. A temperature of 40 °C can be used to increase the sensitivity of the assay, but is more difficult to maintain and control. The slight curvature of the plot below 40 °C and the more pronounced change of the slope above indicate different rate limiting reactions in the assay. The drop in the plot above 50 °C indicates LPMO inactivation. The calculated activation energy *E*_a_ of the reaction is 55.2 kJ mol^−1^. The high activation energy correlates with the low substrate affinity of LPMO for 2,6-DMP and indicates a high contribution of the collision process for the reaction.

In accordance to Bissaro et al. [17], we found that the stability of LPMO is affected by H_2_O_2_. At the low H_2_O_2_ concentration used in the assay (100 µM), no loss of LPMO activity was observed during the assay. However, in titration experiments (Fig. 4a) the biggest achieved total turnover number of *Nc*LPMO9C with H_2_O_2_ was 53. From our observations, we conclude that LPMO is deactivated by H_2_O_2_, especially under turnover conditions.

Control experiments to proof LPMO activity were performed by denaturing LPMO with heat, chelating the copper atom in the active site with EDTA and measuring the activity by exchanging LPMO with Cu(II)SO_4_. Neither the heat inactivated, the EDTA incubated LPMO, nor the Cu(II)SO_4_ activity assays showed a significant activity (Table 2, Additional file 4), which demonstrates that the peroxidase activity is catalyzed by the LPMO holoenzyme.

**Table 2:** 2,6-DMP conversion rates in control reactions at pH 7.5

Specific activities	U g^−1^
*Nc*LPMO9C + 2,6-DMP + H_2_O_2_	32.3 ± 0.9
*Nc*LPMO9C (heat inactivated) + 2,6-DMP + H_2_O_2_	0.2 ± 0.1
*Nc*LPMO9C + 2,6-DMP + H_2_O_2_ + 20 µM EDTA	0.4 ± 0.4
*Nc*LPMO9C + 2,6-DMP (no H_2_O_2_ added)	0.6 ± 0.4
*Nc*LPMO9C + H_2_O_2_ (no 2,6-DMP added)	0.1 ± 0.1
CuSO_4_ + 2,6-DMP + H_2_O_2_	0.6 ± 0.3
CuSO_4_ + 2,6-DMP (no H_2_O_2_ added)	0.5 ± 0.4
2,6-DMP + H_2_O_2_ (no *Nc*LPMO9C added)	0.1 ± 0.1

Concentrations of catalysts/reactants were 0.5 µM *Nc*LPMO9C, 0.5 µM CuSO_4_, 1 mM 2,6-DMP, 100 µM H_2_O_2_

### Assay detection limit

**Fig. 7:**
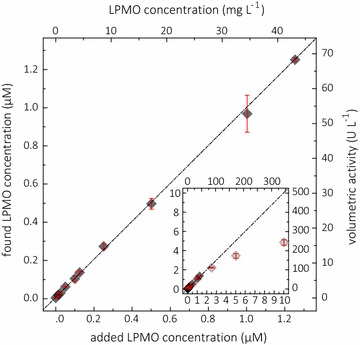
Recovery study for *Nc*LPMO9C concentration. Dark gray diamonds represent the found LPMO concentration plotted against the added LPMO concentration. The activity of different added *Nc*LPMO9C concentrations was measured with the LPMO assay at 30 °C with 1 mM 2,6-DMP, 100 µM H_2_O_2_ in 100 mM succinate/phosphate buffer and the found LPMO concentration was calculated from the measured volumetric activity, the specific LPMO activity of 32.3 U g^−1^, and the molecular mass of *Nc*LPMO9C (34,300 g mol^−1^). A linear range was observed up to a *Nc*LPMO9C concentration of 1.25 µM/42.9 µg mL^−1^. The inset shows the non-linear range with a significant deviation of the found LPMO volumetric activity or recalculated found LPMO concentration. The calculation of the fit is restricted to the linear range. The data are expressed as mean values (± SD), from four independent repeats

To determine the detection limits, a recovery study for LPMO was performed with the assay (Fig. 7). Starting from a LPMO concentration of 0.01 µM, up to 10 µM LPMO was added to the assay. The measured rates from a 300 s reaction (above 1 µM LPMO only initial rates were used) were plotted versus the added LPMO concentration. For LPMO concentrations below 1.25 µM *Nc*LPMO9C, the activity was directly proportional to the enzyme concentration. At enzyme concentrations above 1.25 µM, the activity is no longer proportional and will be underestimated. The measurement range of the assay was determined to be within 0.8 U L^−1^ (corresponding to 0.0125 µM or 0.43 mg L^−1^ *Nc*LPMO9C) and 68 U L^−1^ (corresponding to 1.25 µM or 42.9 mg L^−1^ *Nc*LPMO9C), which is 50 times lower than for the Amplex red assay. The lower limit of the useful range is defined by the limit of detection (LOD), which defines the lower limit of a reliable measurement with respect to the measurement noise (Eq. 1).

1 $${\text{LOD }} = {\text{ LOB }} + \, 1.645 \, \times {\text{ SD }}\left({{\text{lowest}}\;{\text{measurable}}\;{\text{LPMO}}\;{\text{concentration}}} \right)$$

For the limit of the blank (LOB), 24 measurements of the standard assay without LPMO were performed and calculated according to Eq. 2:

2 $${\text{LOB }} = {\text{ mean}}\;{\text{value}}\;{\text{of}}\;24\;{\text{blank}}\;{\text{reactions }} + \, 1.645 \, \times {\text{ SD }}\left( {24\;{\text{blank}}\;{\text{reactions}}} \right)$$

The LOD was determined from 12 different LPMO concentrations, which were measured with the LPMO assay in four, fully randomized technical repeats. Assuming a Gaussian distribution, 95% represents the observed LOB values or LOD low-concentration sample values that exceed the defined LOB, respectively. The remaining 5% of blank values are false positive and only 5% of the low-concentration samples will produce values below the LOB. When using 1.645 × SD, no more than 5% of the values should be less than the LOB [36]. The LOD is the lowest LPMO concentration that can be discriminated from the blank reading with a significance of 0.99 and was calculated to be 0.0125 µM (0.43 µg mL^−1^) of *Nc*LPMO9C.

### LPMO activity assay protocol

Based on the obtained data, we suggest the following assay protocol to test the peroxidase-like activity of LPMO:

Step 1: Prepare a 116 mM sodium succinate/phosphate buffer (or another suitable buffer) by titrating the pH to 6.0 or (for enhanced assay sensitivity) to pH 7.5. The final concentration of the buffer in the cuvette will be diluted to 100 mM. Also prepare a 10 mM 2,6-DMP stock solution (for increased sensitivity at pH 6.0 prepare a 100 mM stock solution) in highly pure water and a 5 mM H_2_O_2_ stock solution in highly pure water. All solutions should be stored separately and used within 12 h.
Step 2: Take 1 mL of sample from the culture supernatant and centrifuge for 3 min at 6000×*g* to remove cells and other solids. Carefully remove 500 µL of the clear supernatant from the sediment and transfer into a clean vial. If taken from a clear solution, the sample needs no centrifugation and a smaller volume suffices. Store on ice until use.
Step 3: Transfer 860 μL of buffer, 100 μL of 2,6-DMP stock solution, and 20 μL of the H_2_O_2_ stock solution into a cuvette and mix the solutions well. Incubate the cuvette for 15 min at 30 °C before continuing with Step 4.
Step 4: Transfer the cuvette into a thermostated sample holder of the spectrophotometer and add 20 μL of properly diluted LPMO. If the LPMO activity is low, higher sample volumes can be used, but the buffer volume and ionic strength have to be adapted accordingly. Measure the increase of absorbance at 469 nm for 300 s and use the correct enzyme factor based on the sample volume, the enzyme dilution, and the molar absorption coefficient of coerulignone (*ε*_469_ = 53,200 M^−1^ cm^−1^) to calculate the peroxidase activity of LPMO.

## Conclusions

The validated LPMO assay procedure is an easy method to quickly follow recombinant LPMO production and purification and is 50 times more sensitive than the previously published Amplex red assay. The 2,6-DMP assay has the potential to broadly be used to enhance the performance of LPMO production and to achieve higher yields and purer LPMO preparations after purification by allowing a more precise fractionation and pooling of the enzyme. The assay can also be used to determine non-catalytic enzyme properties such as thermal unfolding of substrate binding measurements where the peroxidase activity is only used to discriminate active from inactive LPMO molecules or to measure a fraction of LPMO in solution versus the LPMO fraction bound to a polymeric substrate.

## Methods

### Materials and enzymes

All chemicals were of the highest purity grade available and were purchased from Sigma-Aldrich unless stated otherwise. Methanol was purchased from Merck, pyrocatechol from Fluka, and hydrocoerulignone from MP Biomedicals. The lytic polysaccharide monooxygenases *Nc*LPMO9C (Accession Number EAA36362.1) and *Nc*LPMO9F (CAD70347.1) from *Neurospora crassa* and LPMO from *Myriococcum thermophilum* (Myrth2p4_000358 sequence deposited on http://www.fungalgenomics.ca) were recombinantly expressed in *Pichia pastoris* X-33 according to Kittl et al. [16]. Production in a 5-L laboratory fermenter and column purification were also performed according to this publication. The purity of the enzyme was verified by SDS-PAGE. Laccase from *Botrytis aclada* was produced in a 5-L fermenter using *Pichia pastoris* as expression host and column chromatography according to Kittl et al. [37]. Horseradish peroxidase was purchased from Sigma-Aldrich and used without further purification.

### Measurement of LPMO concentration and hydrogen peroxide concentration

The LPMO concentration was determined in a 0.3-mm quartz cuvette from the absorption at 280 nm using molar absorption coefficients and molecular masses of *Nc*LPMO9C *ε*_280_ = 46,910 M^−1^ cm^−1^, 34,300 g mol^−1^; *Nc*LPMO9F *ε*_280_ = 51,130 M^−1^ cm^−1^, 23,200 g mol^−1^; and Myrth2p4_000358 *ε*_280_ = 44,140 M^−1^ cm^−1^, 22,515 g mol^−1^. The H_2_O_2_ concentration was determined in a 10-mm quartz cuvette from the absorption at 240 nm using a molar absorption coefficient of *ε*_240_ = 43.6 M^−1^ cm^−1^.

### Screening of chromogenic substrates

*Nc*LPMO9C was used to probe different phenolic compounds with and without H_2_O_2_ in the presence of molecular oxygen to determine if H_2_O_2_ increases the reaction rate. All experiments were performed in 100 mM succinate/phosphate buffer at pH 6.0. The phenolic compounds were dissolved in water (sinapic acid in methanol, final assay concentration of methanol 2%; hydrocoerulignone in DMSO, final assay concentration of DMSO 2%) and transferred into a cuvette containing 100 mM buffer, 2 µM LPMO, 100 µM H_2_O_2_, and 0.2 mM of the phenol (final concentrations). In reference experiments not containing H_2_O_2_, water was added instead of the H_2_O_2_ solution. Oxidation of the phenols in the presence or absence of H_2_O_2_ was followed for up to 5000 s in a diode-array spectrometer using quartz cuvettes.

### pH optimum of LPMO activity

*Nc*LPMO9C was used to measure enzymatic activity with final concentrations of 100 mM buffer, 100 µM H_2_O_2_, 25 mM DMP, and 0.10–3.75 µM *Nc*LPMO9C (for histidine buffer a 30 µM *Nc*LPMO9C was used to measure activity more accurately). For the whole range of a buffer, the same LPMO concentration was used, and the concentration was only adjusted between different buffers to remain in a measurable range of activity. The anionic buffers were adjusted with sodium hydroxide and the cationic buffers with hydrochloric acid. All measurement were performed at 30 °C and the change in absorbance at 469 nm was followed in a Perkin Elmer Lambda 35 UV/Vis spectrophotometer in triplicates. From pH 3.5 to 7.5, it was not necessary to measure blank reactions without *Nc*LPMO9C, but starting from pH 8.0, a color formation by auto-oxidation of 2,6-DMP is observed. Therefore, it was necessary to measure blank reactions and subtract their slope from the corresponding measurement containing *Nc*LPMO9C. The slope of the initial, steady-state rate was used to calculate the specific activity by using the molar absorption coefficient of coerulignone (*ε*_469_ = 53,200 M^−1^ cm^−1^) and the *Nc*LPMO9C concentration. The effect of the buffer concentration was measured with the buffer systems sodium succinate/phosphate and pyridine/imidazole chloride at concentrations between 5 and 100 mM.

### Control experiments

To probe the described LPMO reaction, we tested the effect of EDTA, heat treatment, the absence of H_2_O_2_, 2,6-DMP, LPMO, and the presence of copper(II) sulfate instead of LPMO. LPMO was added to the solution with final concentrations of 1 mM DMP and 100 µM H_2_O_2_ and 0.5 µM LPMO at 30 °C and measured for 300 s in a diode-array spectrometer using plastic cuvettes. The reaction was followed at 469 nm and the slope and specific activity was calculated. Blank reactions were performed, but omitting LPMO or 2,6-DMP or H_2_O_2_. To denature LPMO by heat, LPMO was added to a final concentration of 0.52 µM to the buffer and incubated for 1 h at 100 °C. To remove the copper atom from the active site of LPMO, we used 1 mM (final concentration) EDTA and incubated 25 µM LPMO for 30 min at 25 °C. Afterwards, the loss of activity was verified by an activity assay. Reactions with 0.5 µM CuSO_4_ were performed at standard conditions, but without LPMO.

### LPMO activity assay

The suggested standard conditions for the LPMO activity assay are a temperature of 30 °C, an H_2_O_2_ concentration of 100 µM, a 2,6-DMP concentration of 1.0 mM, a 100 mM sodium succinate/phosphate buffer at pH 7.5 for maximum robustness and sensitivity or a 2,6-DMP concentration of 10.0 mM at pH 6.0 to account more physiological conditions for some LPMOs, and a reaction time of 300 s. One unit of LPMO activity is defined as the conversion of 2 µmol 2,6-DMP or the formation of 1 µmol coerulignone (*ε*_469_ = 53,200 M^−1^ cm^−1^) per min under reaction conditions.

### LPMO activity assay under anaerobic conditions

Experiments in the absence of oxygen were carried out in a glove box (Whitley DG250, Don Whitley Scientific, Shipley, UK) which was continuously flushed with a nitrogen/hydrogen mixture (90/10). Residual oxygen was removed with a built-in palladium catalyst and the generated water vapor was absorbed on silica gel. Absorption was recorded with an Agilent 8453 UV–Vis spectrophotometer equipped with a photodiode array detector. All chemicals and reagents used within the glove box were extensively degassed by applying alternate cycles of vacuum and nitrogen pressure. Experiments were carried out in stirred cuvettes. Otherwise, the experimental setup was identical to the measurements carried out at ambient conditions. Experiments were carried out in three independent repeats.

### Kinetic constants of LPMO and temperature effects

Kinetic constants were determined in 100 mM succinate/phosphoric acid buffer at pH 6.0 and 7.5. All experiments were performed in triplicates. The resulting curves were fitted to the Michaelis–Menten equation by non-linear least-square regression using *SigmaPlot* 12.5 (Systat Software, Chicago, Illinois, USA). For measurements performed at pH 6.0 2 µM, LPMO was used and for measurements at pH 7.5, 0.125 µM LPMO was used. To test the effect of the reaction temperature, the standard assay was preincubated at temperatures between 25 and 60 °C and 0.5 µM LPMO was added to start the reaction in the photometer.

### Limit of detection

To determine the lowest concentration of LPMO which can be measured, we determined the limit of detection (LOD) for the standard LPMO assay according to [36]. Twenty-four blank reactions without LPMO were measured to calculate the limit of blank (LOB). For the LOD measurement, four independent LPMO dilution series were prepared and measured in a completely randomized measurement scheme, which was prepared using the RAND function in *Microsoft Excel 2016* (Microsoft Cooperation, Redmond, WA, USA). Quadruplets were measured to calculate the specific activity. A 6-time higher volumetric activity compared to the standard error of the blank was used to determine the LOD. To recalculate the LPMO concentration, the average activity of all measurements in the linear range was used.

### Reactant stoichiometry and molar absorption coefficients of 2,6-DMP and hydrocoerulignone

*Nc*LPMO9C, laccase, and horseradish peroxidase activity on 2,6-DMP was compared. The maximally formed absorption at 469 nm of reaction products at pH 6.0 and 7.5 in 10 mM sodium succinate/phosphate buffer was determined for all three enzymes and the molar absorption coefficient for 2,6-DMP and hydrocoerulignone was calculated. Final concentrations for of 2.5–50 µM H_2_O_2_ with 1.0 mM 2,6-DMP or 200 µM hydrocoerulignone and 2.5–50 µM 2,6-DMP or 1.25–10 µM hydrocoerulignone with 100 µM H_2_O_2_ were used and converted with 0.38 µM *Nc*LPMO9C, 0.2 µM laccase, and 0.02 µM HRP. In case of laccase no H_2_O_2_ was added. The molar absorption coefficient of coerulignone was calculated from experiments of all three enzymes considering only substrate concentrations in the linear range.

### Cyclic voltammetry

Measurements were performed at 30 °C using a 4-mL electrochemical cell. The system setup involved a rotating disk electrode made from glassy carbon working electrode, a Ag|AgCl (3 M) reference electrode, a platinum counter electrode, an Autolab Rotator (RDE80739), an Autolab controller, and an Autolab potentiostat (PBSTAT204). The system was controlled using the NOVA 1.11 program from Autolab. Before measurements, the glassy carbon disk electrode was polished with an aluminum oxide suspension (Buehler; Master Prep Polishing Suspension, 0.05 μm). The rotating disk electrode was set to 250 rpm before starting the cyclic voltammetry measurement between 0 and 600 mV vs. the reference electrode with scan rate of 3 mV s^−1^. For the measurement of individual 2,6-DMP concentrations, 5 mL of a 100 mM sodium phosphate buffer, pH 6.0, was added to the cell. The buffer was left to equilibrate the system for 5 min. Different concentrations (0.03, 0.1, 0.3, 1.0, 5.0, and 20.0 mM) of 2,6-DMP were added to the cell, and after mixing for 5 min the measurements were started. The same procedure was performed for measurements at pH 8.0 using 100 mM sodium phosphate buffer, pH 8.0.

## Additional files

**Additional file 1.** Chromogenic substrates screened for activity with *Nc*LPMO9C.

**Additional file 2.** Spectra of the oxidation of sinapic acid, gallic acid, and pyrocatechol by *Nc*LPMO9C.

**Additional file 3.** Determination of 2,6-DMP oxidation potentials by cyclic voltammetry.

**Additional file 4.** Increase of the 2,6-DMP absorbance at 469 nm in control experiments.

## Abbreviations

PPD: 1,4-phenylenediamine
ABTS: 2,2′-azino-bis(3-ethylbenzthiazoline-6-sulfonic acid)
2,6-DMP: 2,6-dimethoxyphenol
DMB: 3,3′-diaminobenzidine
hydrocoerulignone: 3,3′,5,5′-tetramethoxy-4,4′-dihydroxybiphenyl
coerulignone: 3,3′,5,5′-tetramethoxydiphenoquinone
TMB: 3,3′,5,5′-tetramethylbenzidine
CDH: cellobiose dehydrogenase
CAD: charged aerosol detection
CuSO_4_: copper sulfate
DP: degree of polymerization
DMSO: dimethylsulfoxide
ESI-MS: electrospray ionization mass spectrometry
EDTA: ethylenediaminetetraacetic acid
FRET: fluorescence resonance energy transfer
HPAEC: high performance anion exchange chromatography
HRP: horseradish peroxidase
HCl: hydrogen chloride
H_2_O_2_: hydrogen peroxide
HILIC: hydrophilic interaction liquid chromatography
LOB: limit of blank
LOD: limit of detection
LPMO: lytic polysaccharide monooxygenase
MALDI-TOF: matrix-assisted laser desorption ionization-time of flight mass spectrometry
PACE: polysaccharide analysis by carbohydrate gel electrophoresis
PASC: phosphoric acid swollen cellulose
PGC: porous graphitized carbon
NaOH: sodium hydroxide
SD: standard deviation
TMPD: tetramethyl-*p*-phenylendiamine
UHPLC: ultra high performance liquid chromatography
