# Supplementary material for: A fast and sensitive activity assay for lytic polysaccharide monooxygenase
Source: Biotechnol Biofuels. 2018 Mar 23;11:79. doi: 10.1186/s13068-018-1063-6 (PMC5865291; doi:10.1186/s13068-018-1063-6)
Supplement: Supplementary file 1 — Additional file 1. Chromogenic substrates screened for activity with NcLPMO9C. [file 13068_2018_1063_MOESM1_ESM.pdf]

Additional file 1. Chromogenic substrates screened for activity with *Nc*LPMO9C

Name	Comment
4-Aminophenol	Very instable; low reaction rate, no difference with H_2O_2
4-Nitrophenol	Very instable; low reaction rate, no difference with H_2O_2
ABTS	No change in spectra
Acetosyringone	No change in spectra
DMB	Very instable
Guaiacol	No change in spectra
PPD	Very instable; low reaction rate, no difference with H_2O_2
Pyrogallol	Low reaction rate, no difference with H_2O_2
Syringaldehyde	No change in spectra
Syringic acid	No change in spectra
TMB	Very instable; low reaction rate, no difference with H_2O_2
TMPD	Very instable; low reaction rate, no difference with H_2O_2
