# Supplementary material for: A fast and sensitive activity assay for lytic polysaccharide monooxygenase
Source: Biotechnol Biofuels. 2018 Mar 23;11:79. doi: 10.1186/s13068-018-1063-6 (PMC5865291; doi:10.1186/s13068-018-1063-6)
Supplement: Supplementary file 2 — Additional file 2. Spectra of the oxidation of sinapic acid, gallic acid, and pyrocatechol by NcLPMO9C. [file 13068_2018_1063_MOESM2_ESM.pdf]

Additional file 2. Spectra of the oxidation of sinapic acid, gallic acid, and pyrocatechol by *Nc*LPMO9C.

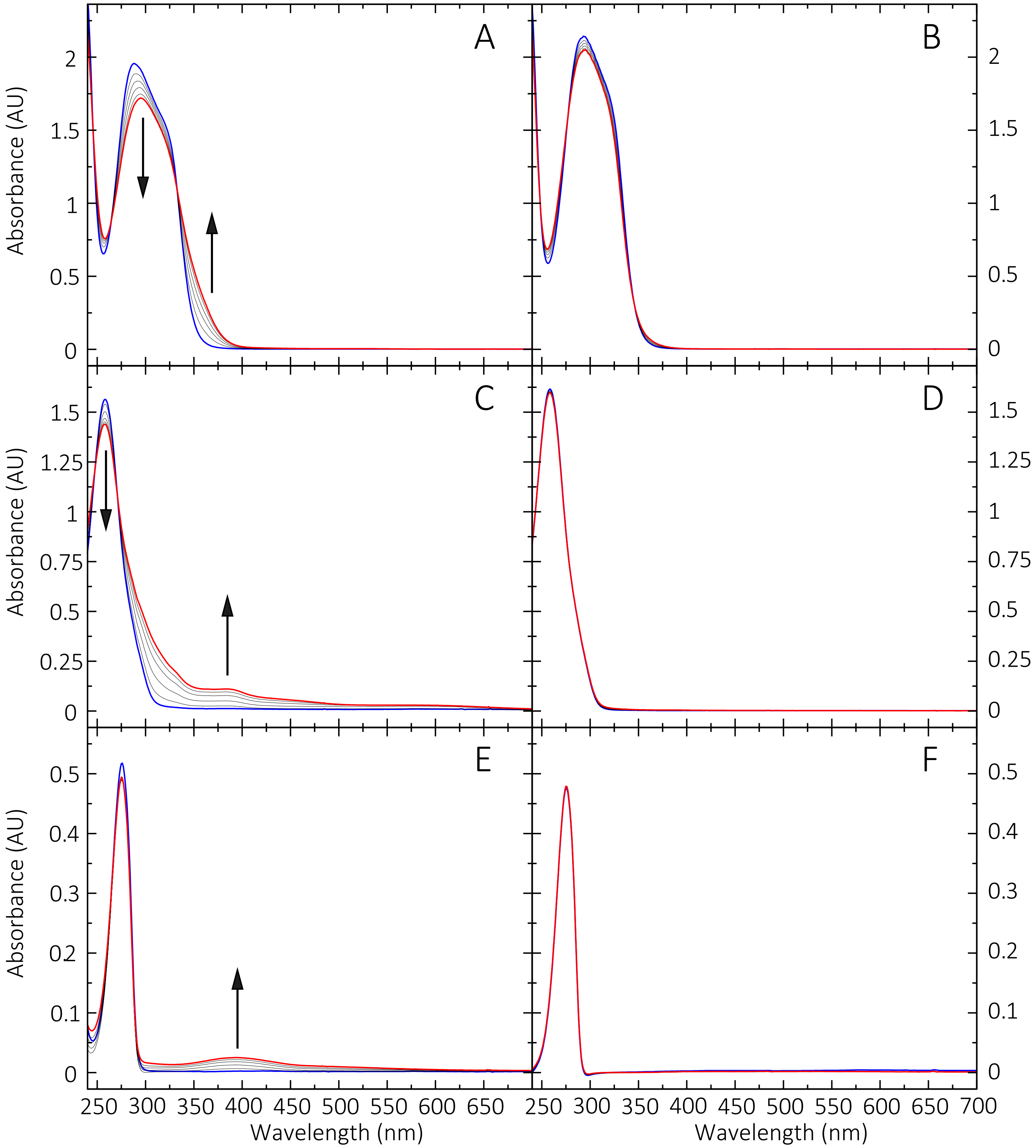

Spectra of phenolic compounds with and without H_2O_2 in presence of O_2. (**A**) Sinapic acid with H_2O_2 (**B**) in presence of O_2 but without H_2O_2 measured over 5000 s. (**C**) Gallic acid with H2O2 (**D**) in presence of O_2 but without H_2O_2 measured over 2500 s. (**E**) Pyrocatechol with H_2O_2 (**F**) in presence of O_2 but without H_2O_2 measured over 2500 s. Experimental conditions: 100 mM sodium succinate/phosphate buffer, pH 6.0; 2 µM *Nc*LPMO9C, ~250 µM O_2, and if indicated 100 µM H_2O_2.
