# Supplementary material for: A fast and sensitive activity assay for lytic polysaccharide monooxygenase
Source: Biotechnol Biofuels. 2018 Mar 23;11:79. doi: 10.1186/s13068-018-1063-6 (PMC5865291; doi:10.1186/s13068-018-1063-6)

Additional file 3. Determination of 2,6-DMP oxidation potentials by cyclic voltammetry. The potential was measured for different 2,6-DMP concentrations at pH 6.0 and 8.0.

2,6-DMP concentration (mM)	*0.03*	*0.1*	*0.3*	*1.0*	*5.0*	*20.0*
pH 6.0	305	258	215	150	105	95
pH 8.0	200	150	120	25	22	10

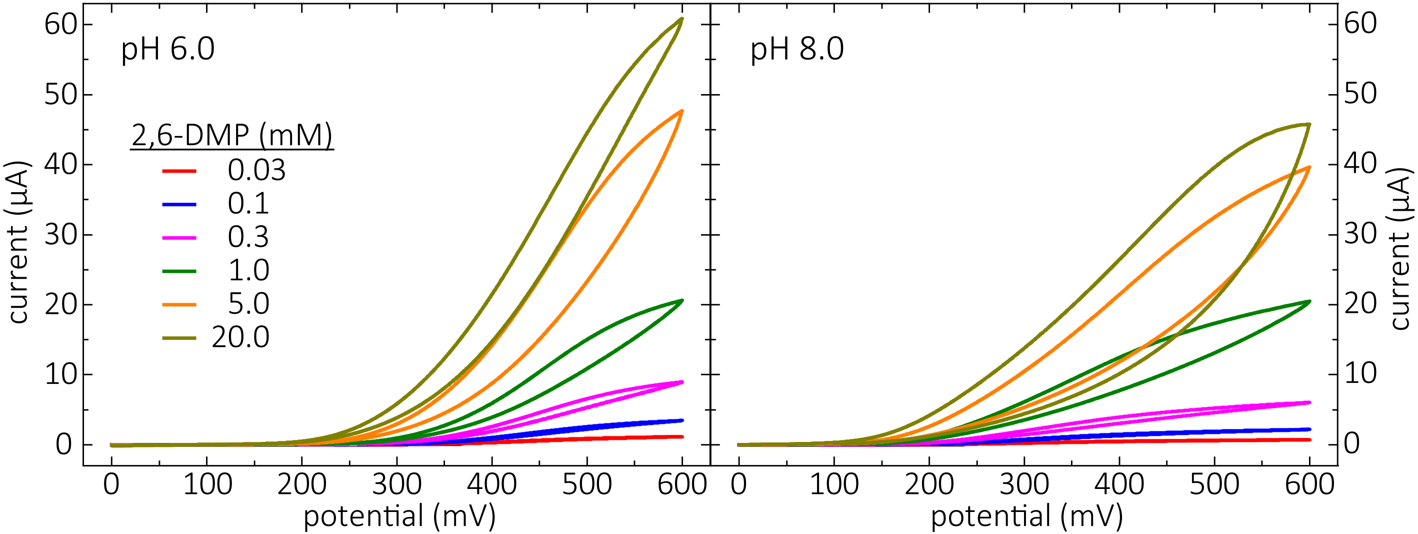

Supplement: Supplementary file 3 — Additional file 3. Determination of 2,6-DMP oxidation potentials by cyclic voltammetry. [file 13068_2018_1063_MOESM3_ESM.pdf]
