# Supplementary figures and images for: A fast and sensitive activity assay for lytic polysaccharide monooxygenase
Source: Biotechnol Biofuels. 2018 Mar 23;11:79. doi: 10.1186/s13068-018-1063-6 (PMC5865291; doi:10.1186/s13068-018-1063-6)

**Additional file 4.** Increase of the 2,6-DMP absorbance at 469 nm in control experiments.

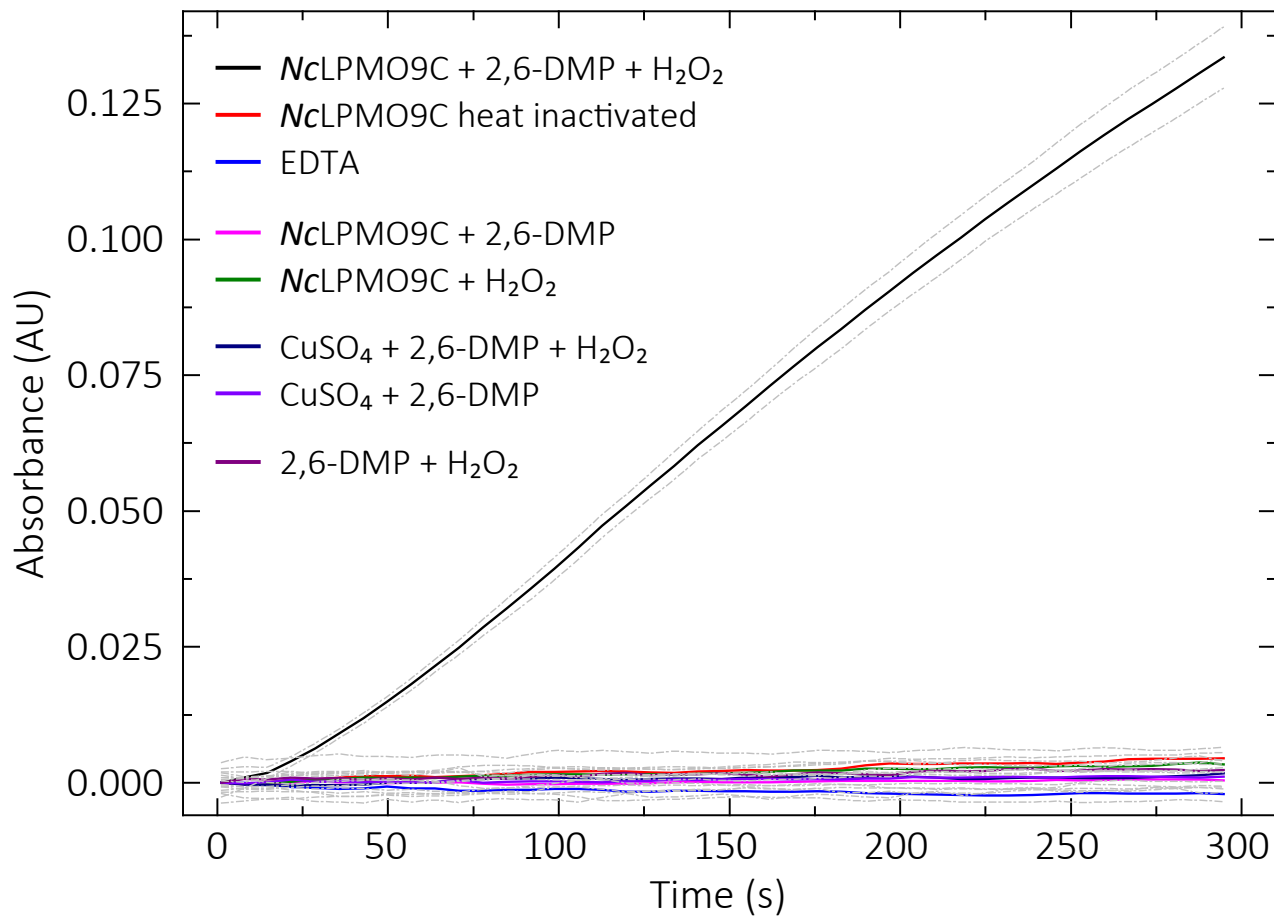

Supplement: Supplementary file 4 — Additional file 4. Increase of the 2,6-DMP absorbance at 469 nm in control experiments. [file 13068_2018_1063_MOESM4_ESM.pdf]
